# The Role of PPARs in Placental Immunology: A Systematic Review of the Literature

**DOI:** 10.1155/2013/970276

**Published:** 2013-03-10

**Authors:** Stefan Hutter, Julia Knabl, Ulrich Andergassen, Udo Jeschke

**Affiliations:** Department of Gynecology and Obstetrics, Ludwig Maximilians University of Munich, Maistraße 11, 80337 Munich, Germany

## Abstract

Pregnancy is a state of immunotolerance, and pregnancy outcome is strongly linked to the correct activation and balancing of the maternal immune system. Besides abortion as possible result of improper early pregnancy development, other pregnancy associated conditions like preeclampsia (PE), intrauterine growth retardation (IUGR), preterm labour, or gestational diabetes mellitus (GDM) are linked to immunologic overactivation and dysregulation. Both the innate and the adaptive immune system, and therefore B and T lymphocytes, natural killer cells (NK), macrophages and dendritic cells (DCs) are all involved in trophoblast invasion, pregnancy maintenance, and development of pregnancy disorders. Peroxisome proliferator activated receptors (PPARs) are nuclear transcription factors with three known isotypes: PPAR*α*, PPAR**β**/**δ**, and PPAR**γ**. They are expressed in most human organs and their function extends from regulating metabolism, homeostasis, and carcinogenesis to immune response. In the recent years, PPARs have been identified in most reproductive tissues and in all lines of immune cells. Only in few cases, the role of PPARs in reproductive immunology has been elucidated though the role of PPARs in immune answer and immunotolerance is evident. Within this paper we would like to give an update on today's knowledge about PPARs and immune cells in reproduction and highlight interesting interferences in regard of future therapeutic targets.

## 1. Introduction

Trophoblast invasion at the beginning of pregnancy development is often described in the context of pregnancy complications. The underlying pathophysiology includes elevated macrophage populations hampering trophoblast invasion and inducing trophoblast apoptosis [1, 2]. As another, even bigger part of the innate immune response decidual natural killer (dNK) cells have been identified in promoting trophoblast invasion [3] and reduced numbers of dNK have been reported associated with IUGR [4]. The components of the adaptive immune system present at the fetomaternal interface as the site of immunologic tolerance plays an important role in balancing the maternal immune response to the fetal allograft. For many years a simplified approach to this complex process of immunotolerance was postulated. Thus pregnancy was regarded as a phenomenon of T helper cells subgroup 2 (Th2) going along with inhibition of T helper cells subgroup 1 (Th1) and their cytotoxic effects [5]. However the results of the last years have shown that cytokines associated with Th1 are closely linked to a positive pregnancy outcome [6]. Besides this formerly used concept of Th1 and Th2 immune response additional Th17 cells and regulatory T cells (T reg) have been described to show their importance concerning pregnancy outcome. In PE T reg have been shown to be reduced in number [7], whereas regular pregnancies go along with increased numbers of T reg [8]. The importance of the adaptive and innate immune system at the fetomaternal interface, that is, the extra villous trophoblast (EVT), is obvious not just in terms of late term pregnancy complications such as PE or IUGR but even more when talking about miscarriages and maintaining early pregnancy. In this context not only T cell regulation has been described but also macrophage population and its dynamic polarization seem to play a role concerning the success of early pregnancy [9]. 

As part of the nuclear hormone receptor (NHR) superfamily peroxisome proliferator activated receptors have been discovered in the year 1990. They act as ligand activated transcription factors binding to DNA as heterodimers with retinoid X receptor (RXR) inducing transcription of various genes [10]. Their function is triggered by the shape of the ligand binding domain and by coactivator and corepressor proteins. Among those are free fatty acids and eicosanoids that are, mostly prostaglandins and leukotrienes [11]. Originally PPARs were mostly associated with metabolic aspects as fatty acid transport or oxidation [12]. However, PPARs have been identified to play an important role in carcinogenesis, homeostasis, and immune system. Both the innate and the adaptive immune system, are strongly influenced by PPAR activation triggering macrophages and other leukocyte populations such as lymphocytes and dendritic cells [13, 14]. Concerning placenta as the spot of feto-maternal immuno-tolerance PPARs and their effect on macrophage population and polarization as well as lymphocyte differentiation have come to scientific interest and basic research on this aspect of placental immunology has been done over the past years. Within this paper we would like to show interesting findings on the field of PPAR research in placental tissue and point out recent findings of PPAR influence on macrophages and T lymphocytes in general thus encouraging further research in the field of PPAR and placental immune response.

## 2. Material and Methods

By searching PubMed 942 relevant studies up to November 2012 were identified. Only publications in English language were selected. Following keywords were used individually and in combination. PPAR, macrophages, T lymphocytes, B lymphocytes, placenta, and reproduction. A systematic literature review was conducted using the literature provided and based on our findings and experience in PPAR function in female reproduction. Relevant information on PPAR and macrophages and lymphocytes in reproductive context were gathered to offer an updated summary on this topic.

## 3. PPARs and Reproduction

By now three isotypes of PPAR, that is, PPAR*α*, PPPAR*β*/*δ*, and PPAR*γ* have been described [15]. All three are expressed in human placenta and different functions have been attributed to them. Interestingly concentration and function of PPAR isotypes in placenta change throughout pregnancy in humans and in animal models [16, 17]. For PPAR*γ* and PPAR*β*/*δ* a role in trophoblast differentiation, trophoblast invasion, and decidualization has been established [17, 18]. Also for PPAR*α*  there is evidence for its supportive role in placental development and creating an anti-inflammatory placental environment by negatively effecting lipid peroxidation and production of nitric oxide (NO) [19]. Concerning PPAR*β*/*δ* its importance and positive effect on placentation timing and uterine angiogenesis has been shown in the mouse model [20]. PPAR*γ* is by now well established in several aspects of placenta biology such as regulation of trophoblast invasion and early development. It has been shown to modulate the expression of proinflammatory genes such as matrix metalloproteinases [21]. A supposedly detrimental role for PPAR*γ* has been described in the case of human cytomegalovirus (HCMV) as the virus is causing an activation of PPAR*γ* and therefore hampering invasion of the trophoblast [22]. Concluding the preliminary research PPAR*γ* seemingly takes the most important role among the three isotypes in placenta differentiation and immunology. 

### 3.1. PPAR*α*

PPAR*α*  is highly expressed in various organs such as liver and heart, where PPAR*α*  regulates lipid metabolism and anti-inflammatory pathways [23, 24]. So far PPAR*α*  has also been identified in placenta and different fetal organs in postplacentation period [25]. In a rat model changes of PPAR*α*  concentration were shown to appear during the course of pregnancy and its activation was linked to a negative regulation of lipid peroxidation and nitric oxide (NO) production [26]. In mice adverse reproductive outcomes in the context of disrupted carbon-1 metabolism could be attributed to PPAR*α*  effects, which lead to an imbalance in the Th1 : Th2 ratio through increased maternal and fetal interferon *γ* (IFN*γ*) and decreased maternal interleukine 10 (IL-10) [27]. Perturbations in PPAR*α*  caused by a disruption of carbon-1 metabolism showed also anti-inflammatory effects of PPAR*α*  by increasing the production of Th2 cytokines IL-10 and IL-4 and by reducing the production of Th1 specific cytokines like IFN*γ* and IL-2 in a mouse model [28]. 

Contrarily to former statements PPAR alpha seems to have impact on pregnancy outcome by influencing T cell differentiation and therefore T cell specific cytokine production.

### 3.2. PPAR*β*/*δ*

PPAR*β*/*δ*, which is also referred to as PPAR*β* or PPAR*δ*, is encoded by one gene (nuclear receptor subfamily 1, group C, member 2; NR1C2) and belongs to the well-discussed family of PPARs and is highly expressed at the human placenta [29]. Its role in pregnancy development and maintaining pregnancy has been shown for both uterine and embryonic PPAR*β*/*δ* by stating their pivotal roles in placental angiogenesis and on time placentation [30]. PPAR*β*/*δ* null mice showed reduced birth weight and placental defects hereby underlining the importance of PPAR*β*/*δ* for placental function [31]. An in vitro model of trophoblast cells has shown further implications of PPAR*β*/*δ* effects on enzyme expression in trophoblast cells. 11-hydroxysteroid dehydrogenase type 2 (11-HSD2), which is responsible of protecting the fetus from exposure to high levels of maternal glucocorticoid, seems to be repressed by activation of PPAR*β*/*δ*, consecutively causing IUGR. Leaving out in vivo coactivation effects on 11-HSD2, which might influence its expression and function, it is attempting to speculate whether over-expression of PPAR*β*/*δ* is linked to placental pathologies via this pathway [32]. Impact of PPAR*β*/*δ* on inflammation processes has been shown in central nervous system autoimmunity by reducing inflammatory T cells. In both mouse and human immune cells PPAR*β*/*δ* was found to reduce production of IFN*γ* and IL-12 family cytokines and expansion of CD 4+ cells hereby reducing the inflammatory reaction [33]. Concerning miscarriages PPAR*β*/*δ* expression has been shown to be enhanced in miscarried placentas whereas leptin expression appeared to be low [34]. Obviously PPAR*β*/*δ* cannot attribute a clear pro- nor an anti-inflammatory function at this point of time; however its presence and importance in immune response is indisputable.

### 3.3. PPAR*γ*

PPAR*γ* alike the other PPAR isotypes was at first described in its role in metabolic control and homeostasis gaining importance because of its highly efficient ligands, which are currently made use of in diabetes therapy. Further studies have shown its role in trophoblast function and invasion and treatment with PPAR*γ* agonists led to fetal and placental growth restriction in a PPAR*γ* dependent manner [35, 36]. Contrarily rats treated with PPAR*γ* antagonists demonstrated impaired placentation and placental differentiation, thus implying differences in effect of PPAR*γ* activation or blocking according to the stage of pregnancy development [37]. In regard of IUGR and obesity PPAR*γ* expression was found to be increased in human placenta; hereby this upregulation could be interpreted as adaptive response to the IUGR placenta preventing insufficient nutrient supply [38, 39]. Alternatively PPAR*γ* expression could be seen as causative for IUGR and placental failure as it was shown in different studies based on PPAR*γ* specific activation [36, 40]. Mouse knockout models reacted with fetal loss in early pregnancy due to the missing PPAR*γ* expression and showed placental defects [31]. Further studies with continuous PPAR*γ* antagonist treatment in uncomplicated rat pregnancies have provided evidence for the pivotal role of PPAR*γ* in development of PE, as this treatment led to an increase in soluble fms-like tyrosine kinase 1, which is strongly linked to the pathogenesis of PE. Therefore PPAR*γ* might offer a potential therapeutic target for the treatment and prevention of PE [41]. 

 Additionally research on human placentas has been able to show the importance of PPAR*γ* in cytokine production as IL-6, IL-8, and TNF*α*  were reduced by PPAR*γ* ligands troglitazone and 15d-PGJ2 [42]. 

In Macrophages stimulated with IL-4 PPAR*γ* is markedly induced [43] and therefore further investigation was done to elucidate the role of PPAR*γ* in alternative activation of macrophages. In a mouse model setting of high caloric uptake alternative activation of macrophages was not possible without PPAR*γ* expression and therefore affected individuals were more prone to obesity and insulin resistance [44]. 

## 4. PPAR Activation in Macrophages

Among the immune cells regulating early pregnancy development and implantation T cells and NK cells have been essentially described. At the site of trophoblast invasion decidual macrophages are the second most predominant cell line. Producing inflammatory cytokines and presenting antigen they are known to regulate T-cell activation and therefore apoptosis [45]. 

According to today's research findings about macrophages two classical phenotypes are known—M1 as inflammation phenotype and M2 as promotor of immune modulation. Considering pregnancy as state of balancing immune tolerance at the fetomaternal interface M2 phenotype would be expected in regular pregnancy development and M1 would be linked to miscarriage or failure of implantation. As macrophage plasticity has been demonstrated to be dynamic in character this more or less simple model of thought has lost its importance [46]. It seems obvious that macrophages, which are increased in number in cases of spontaneous miscarriage [15], can react to external stimuli and conditions and therefore a more complex function of macrophages in terms of reproductive immunology is considered. Macrophages stimulated by IFN*γ* differentiate into the classically activated (M1) phenotype, thus implying inflammation and activation of T-lymphocytes. On the other hand IL-4 can induce macrophage differentiation into the alternatively activated (M2) phenotype via expression of PPAR*γ* and PPAR gamma activator [47]. By its transrepressive action PPAR*γ* can also stop the nuclear factor kappa B (NF*κ*B) mediated production of pro-inflammatory mediators [48]. Leishmania infection, for example, benefits from infiltrating macrophages and from Leishmania parasites producing PPAR*γ* ligands that lead to inactivation of the destructive inflammation response. Thus PPAR*γ* activation leads to M2 differentiation and consequently allows an immunomodulatory response and chronic stage in this parasitary disease [49]. Further evidence for PPAR*γ* effects on macrophage activation and differentiation comes from a transgenic mouse model. Macrophage specific PPAR*γ* deletion led to diet associated obesity, insulin resistance, and glucose intolerance [44]. The effect of PPAR*γ* activation on macrophage differentiation became even clearer in the field of atherosclerosis, where monocytes developed into enhanced M2 phenotype after PPAR*γ* activation. Additionally these M2 macrophages implemented more pronounced anti-inflammatory components of M1 macrophages. This interference with activated M1 macrophages is transported via negative influence on pro-inflammatory signaling pathways such as activating protein 1 (AP-1), signal transducer activator of transcription (STAT-3) and NF*κ*B [50, 51]. Interestingly this effect of PPAR*γ* is operating mainly in presence of adequate stimulation for monocytes into M2 macrophages such as IL-4 and not in already differentiated M1 phenotypes. This observation in the field of atherosclerosis could be demonstrating limitations to macrophage plasticity. Macrophage plasticity, which is obviously strongly PPAR dependent, has also been shown in pregnancy development and maintenance [52]. Macrophages are highly active in phagocytosis of apoptotic cells in order to reduce autoimmune response to self-antigens during pregnancy. PPAR*δ* has been shown to be highly expressed in such macrophages and can attribute a pivotal role in clearance of apoptotic cells by macrophages. In case of PPAR*δ* deletion in macrophages a decrease in opsonin expression like C1qb and therefore impairment of phagocytosis is the result [53]. 

## 5. PPARs in Other Immune Cells

Various leukocyte populations express PPAR*γ* and therefore a role in immune response of lymphocytes and dendritic cells is suggested for this transcription factor [14]. PPAR*α*  has been described as suppressor of Th1 immunity and promotor of Th2 immunity [27, 28, 54]. As Th1 and Th2 cytokines have contrary effects on human pregnancy T cell differentiation towards Th1 has been identified as one of the major reasons for abortion, IUGR and PE [55, 56]. Decreased levels of maternal PPAR*α*  have been brought into causative context with an imbalance in Th1 : Th2 ratio leading to increased IFN*γ* and reduced maternal IL-10 and therefore implicating miscarriage and abortion [27]. PPAR*β*/*δ* has been proved to be an important negative regulator in central nervous system autoimmune inflammation. Besides of the inhibition of IFN-*γ* and IL-12 family cytokines PPAR*β*/*δ* has been attributed an important role in downregulation CD 4+ T cell population [57]. 

15-Deoxy-Δ-Prostaglandin J2 (15d-PGJ2), which is an endogenous prostaglandin and acts as ligand for chemoattractant receptor-homologous molecule expressed on Th2 cells (CRTH2), has been shown to suppress the expression of NF*κ*B, which leads to a decrease in Th1 cell cytokines IFN*γ* and TNF*α*. Activation via PPAR*γ* and therefore therapeutic approach via PPAR agonists has been ruled out explicitly for this regulatory mechanism in amnion and myometrial cells [58]. Whether 15dPGJ2, which is also a ligand for PPAR*γ*, is furthermore capable of promoting an anti-inflammatory shift in cytokine production via PPAR*γ* is still to be elucidated.

An important part of the adaptive immune system is taken over by B lymphocytes, which differentiate into immunoglobulin-producing plasma cells therefore setting up the basis for humoral immune response [59]. B lymphocytes that have been described quantitatively changed in cases of recurrent abortion, for example, caused by antiphospholipid syndrome [60]. Concerning the role of B lymphocytes on PE pathophysiology extensive research has identified them as pro-inflammatory agents by mass producing of immunoglobulin and therefore promoting hypertensive reaction via angiotensin II type I receptor (AT1-AA) [61]. Depletion of B cells in this context led to lower blood pressure response to placental ischemia and lower levels of TNF*α*  [62]. Expression of PPAR*γ* has been described for normal and malignant B cells and additionally certain types of PPAR ligands were identified to inhibit B cell proliferation [63]. Activated B cells upregulate their expression of PPAR*γ* and beyond this PPAR ligands such as 15d-PGJ2 (endogenous) and rosiglitazone (synthetic) proofed to stimulate B cell proliferation and differentiation. Furthermore PPAR*γ* ligands enhanced the expression of cyclooxygenase 2 (Cox-2) and the plasma cell transcription factor BLIMP-1. Antagonists, however, showed the reverse effect on B cell population [64]. 

NK cells are the most abundant immune cell population at the site of implantation and have been long recognized as important regulators in early pregnancy maintenance partially in cooperation with dendritic cells (DCs) [65]. Underlining this statement DCs have been reported to elevate induction of regulatory T reg due to crosstalk with uterine NK cells and uterine DC to enhance proliferation of NK cells producing IL-10 [66, 67]. Obviously both cell types contribute to regular implantation as depletion mice models for each cell type have shown [68]. Production of IFN*γ* and cytolytic activity are two major functions of NK cells; both functions being essential for innate immunity [69]. PPAR*γ* ligands were reported to influence both functions via PPAR*γ* and without this pathway. The natural PPAR*γ* ligand 15d-PGJ2 and the synthetic ligand ciglitazone both reduce the production of IFN*γ* via PPAR*γ* expression. However cytotoxic activity of NK cell seems not affected by PPAR*γ* expression. Interestingly IFN*γ* levels were decreased by 15d-PGJ2 even without influencing PPAR*γ* expression, suggesting that this effect takes place at a posttranscriptional level [70]. 

DCs are extensively described as antigen presenting cells triggering the T cell immune response and T cell differentiation also in reproductive organs. Their importance in decidualization and vascularization has been shown in depletion models of mice [68]. PPAR*γ* has been found to prevent IL-12 production, which is indispensable for Th1 differentiation and therefore PPAR*γ* might shift naïve T cells to Th2 via its effect on DC [71, 72]. 

## 6. Summary

Placental architecture and function play a crucial role in fetal development and pregnancy maintenance. Correct placental differentiation and trophoblast invasion are highly important preconditions for fetal growth and appear to be altered in several pregnancy complications. PE, IUGR, and GDM are strongly linked to placenta changes [73, 74]. Each of these pregnancy related disorders shares similar risk factors and comes along with an elevated risk for developing the other [75, 76]. Risk factors include obesity, hypertension, previous episodes of GDM, PE or IUGR and a family history of such disorders [77, 78]. Common risk factors allow to draw conclusions on common aspects of pathophysiology highlighting improper placentation, processes of inflammation and elevated levels of cytokines and oxidative stress [79–81]. 

Mostly PPAR*β*/*δ* and PPAR*γ* have been identified in macrophages influencing differentiation and cytokine production to an anti-inflammatory profile. Considering the well described dynamic plasticity of macrophages especially in terms of pregnancy related tolerance PPAR ligands could evolve to an interesting therapeutic agent by promoting the M2 phenotype and therefore improving pregnancy development and reducing glucose intolerance. In analogy to the formerly proposed model of M1 versus M2 distribution of macrophage population the paradigm of pregnancy as Th2 phenomenon has been left. However, the Th1 : Th2 ratio still plays an important role in pregnancy as T cell specific cytokine production is essential for immunotolerance at the fetomaternal interface. PPAR*β*/*δ* and PPAR*γ* have been described as promotor of the anti-inflammatory Th2 differentiation [54, 57]. Besides endogenous PPAR*γ* ligands (15d-PGJ2) other synthetic ligands have been shown to promote an anti-inflammatory cytokine profile in Th1 and Th2 cells; however it remains unclear if all these effects are induced via PPAR activation [58]. The potential effect of PPAR ligands on T cell differentiation would still be a promising approach to the treatment of inflammatory activation in pregnancy. B lymphocytes considering pregnancy disorders mostly associated with PE have been shown to get activated by PPAR agonists and a reverse effect was described for PPAR antagonists [64]. In a similar way NK cells can be influenced by endogenous and synthetic PPAR ligands. However this influence seems to be limited to their cytokine production [70].

Looking at therapeutic strategies in order to prevent loss of pregnancy or miscarriage changing Th1 immune response [82] via PPAR activation could be a reasonable solution. Use of PPAR*γ* agonists could be useful in preventing preterm labour by reducing inflammatory response within the fetal membranes [83]. Also lifestyle and nutrition changes could become recommendable according to PPAR*γ* actions as linoleic acid as component of vegetables, fruits, grains, seeds, and others is easily converted to a ligand of PPAR*γ* by the gut flora in combination with probiotics [84].

Summing up PPARs evidently play an important role in immune cell differentiation and interaction of the innate and adaptive immune system. They offer various opportunities for therapeutic approach assuming that further research is done in order to elucidate their range of effects on immune response reaching from pro- to anti-inflammatory. So far immune cells in reproduction have been focused intensively concerning early and late pregnancy; however, knowledge about PPAR activation effects on immune cells concerning especially pregnancy issues is limited. As PPAR activation of immune cells and the importance of immune cells in pregnancy are both well described further research in this field is very promising in terms of understanding placental immunology and finding therapeutic targets. 

## References

[1] Renaud SJ, Postovit LM, Macdonald-Goodfellow SK, McDonald GT, Caldwell JD, Graham CH (2005). Activated macrophages inhibit human cytotrophoblast invasiveness in vitro. *Biology of Reproduction*.

[2] Reister F, Frank HG, Kingdom JCP (2001). Macrophage-induced apoptosis limits endovascular trophoblast invasion in the uterine wall of preeclamptic women. *Laboratory Investigation*.

[3] Hiby SE, Walker JJ, O’Shaughnessy KM (2004). Combinations of maternal KIR and fetal HLA-C genes influence the risk of preeclampsia and reproductive success. *Journal of Experimental Medicine*.

[4] Williams PJ, Bulmer JN, Searle RF, Innes BA, Robson SC (2009). Altered decidual leucocyte populations in the placental bed in pre-eclampsia and foetal growth restriction: a comparison with late normal pregnancy. *Reproduction*.

[5] Wegmann TG, Lin H, Guilbert L, Mosmann TR (1993). Bidirectional cytokine interactions in the maternal-fetal relationship: is successful pregnancy a TH2 phenomenon?. *Immunology Today*.

[6] McCracken SA, Gallery E, Morris JM (2004). Pregnancy-specific down-regulation of NF-kappa B expression in T cells in humans is essential for the maintenance of the cytokine profile required for pregnancy success. *The Journal of Immunology*.

[7] Sasaki Y, Darmochwal-Kolarz D, Suzuki D (2007). Proportion of peripheral blood and decidual CD4^+^CD25^bright^ regulatory T cells in pre-eclampsia. *Clinical and Experimental Immunology*.

[8] Tilburgs T, Roelen DL, Van Der Mast BJ (2008). Evidence for a selective migration of fetus-specific CD4^+^CD25^bright^ regulatory T cells from the peripheral blood to the decidua in human pregnancy. *The Journal of Immunology*.

[9] Guenther S, Vrekoussis T, Heublein S (2012). Decidual macrophages are significantly increased in spontaneous miscarriages and over-express fasL: a potential role for macrophages in trophoblast apoptosis. *International Journal of Molecular Sciences*.

[10] Semple RK, Chatterjee VKK, O’Rahilly S (2006). PPAR*γ* and human metabolic disease. *The Journal of Clinical Investigation*.

[11] Yu S, Reddy JK (2007). Transcription coactivators for peroxisome proliferator-activated receptors. *Biochimica et Biophysica Acta*.

[12] Beaven SW, Tontonoz P (2006). Nuclear receptors in lipid metabolism: targeting the heart of dyslipidemia. *Annual Review of Medicine*.

[13] Chinetti G, Fruchart JC, Staels B (2000). Peroxisome proliferator-activated receptors (PPARs): nuclear receptors at the crossroads between lipid metabolism and inflammation. *Inflammation Research*.

[14] Standiford TJ, Keshamouni VG, Reddy RC (2005). Peroxisome proliferator-activated receptor-{gamma} as a regulator of lung inflammation and repair. *Proceedings of the American Thoracic Society*.

[15] Michalik L, Desvergne B, Dreyer C, Gavillet M, Laurini RN, Wahli W (2002). PPAR expression and function during vertebrate development. *International Journal of Developmental Biology*.

[16] Lappas M, Rice GE (2009). Transcriptional regulation of the processes of human labour and delivery. *Placenta*.

[17] Holdsworth-Carson SJ, Lim R, Mitton A (2010). Peroxisome proliferator-activated receptors are altered in pathologies of the human placenta: gestational diabetes mellitus, intrauterine growth restriction and preeclampsia. *Placenta*.

[18] Tarrade A, Schoonjans K, Pavan L (2001). PPAR*γ*/RXR*α* heterodimers control human trophoblast invasion. *Journal of Clinical Endocrinology and Metabolism*.

[19] Martínez N, Kurtz M, Capobianco E, Higa R, White V, Jawerbaum A (2011). PPAR*α* agonists regulate lipid metabolism and nitric oxide production and prevent placental overgrowth in term placentas from diabetic rats. *Journal of Molecular Endocrinology*.

[20] Wang H, Xie H, Sun X (2007). Stage-specific integration of maternal and embryonic peroxisome proliferator-activated receptor *δ* signaling is critical to pregnancy success. *The Journal of Biological Chemistry*.

[21] Tarrade A, Schoonjans K, Pavan L (2001). PPAR*γ*/RXR*α* heterodimers control human trophoblast invasion. *Journal of Clinical Endocrinology and Metabolism*.

[22] Rauwel B, Mariamé B, Martin H (2010). Activation of peroxisome proliferator-activated receptor gamma by human cytomegalovirus for *De Novo* replication impairs migration and invasiveness of cytotrophoblasts from early placentas. *Journal of Virology*.

[23] Belfort R, Berria R, Cornell J, Cusi K (2010). Fenofibrate reduces systemic inflammation markers independent of its effects on lipid and glucose metabolism in patients with the metabolic syndrome. *Journal of Clinical Endocrinology and Metabolism*.

[24] Lefebvre P, Chinetti G, Fruchart JC, Staels B (2006). Sorting out the roles of PPAR alpha in energy metabolism and vascular homeostasis. *The Journal of Clinical Investigation*.

[25] Wang Q, Fujii H, Knipp GT (2002). Expression of PPAR and RXR isoforms in the developing rat and human term placentas. *Placenta*.

[26] Martínez N, Kurtz M, Capobianco E, Higa R, White V, Jawerbaum A (2011). PPAR*α* agonists regulate lipid metabolism and nitric oxide production and prevent placental overgrowth in term placentas from diabetic rats. *Journal of Molecular Endocrinology*.

[27] Mikael LG, Pancer J, Wu Q, Rozen R (2012). Disturbed one-carbon metabolism causing adverse reproductive outcomes in mice is associated with altered expression of apolipoprotein AI and inflammatory mediators PPAR*α*, interferon-*γ*, and interleukin-10. *Journal of Nutrition*.

[28] Yessoufou A, Hichami A, Besnard P, Moutairou K, Khan NA (2006). Peroxisome proliferator-activated receptor *α* deficiency increases the risk of maternal abortion and neonatal mortality in murine pregnancy with or without diabetes mellitus: modulation of T cell differentiation. *Endocrinology*.

[29] Mukherjee R, Jow L, Croston GE, Paterniti JR (1997). Identification, characterization, and tissue distribution of human peroxisome proliferator-activated receptor (PPAR) isoforms PPAR*γ*2 versus PPAR*γ*1 and activation with retinoid X receptor agonists and antagonists. *The Journal of Biological Chemistry*.

[30] Wang H, Xie H, Sun X (2007). Stage-specific integration of maternal and embryonic peroxisome proliferator-activated receptor *δ* signaling is critical to pregnancy success. *The Journal of Biological Chemistry*.

[31] Barak Y, Liao D, He W (2002). Effects of peroxisome proliferator-activated receptor *δ* on placentation, adiposity, and colorectal cancer. *Proceedings of the National Academy of Sciences of the United States of America*.

[32] Julan L, Guan H, Van Beek JP, Yang K (2005). Peroxisome proliferator-activated receptor *δ* suppresses 11*β*-hydroxysteroid dehydrogenase type 2 gene expression in human placental trophoblast cells. *Endocrinology*.

[33] Dunn SE, Bhat R, Straus DS (2010). Peroxisome proliferator-activated receptor *δ* limits the expansion of pathogenic Th cells during central nervous system autoimmunity. *Journal of Experimental Medicine*.

[34] Toth B, Bastug M, Scholz C (2008). Leptin and peroxisome proliferator-activated receptors: impact on normal and disturbed first trimester human pregnancy. *Histology and Histopathology*.

[35] Schaiff WT, Knapp FF, Barak Y, Biron-Shental T, Nelson DM, Sadovsky Y (2007). Ligand-activated peroxisome proliferator activated receptor *γ* alters placental morphology and placental fatty acid uptake in mice. *Endocrinology*.

[36] Tarrade A, Schoonjans K, Pavan L (2001). PPAR*γ*/RXR*α* heterodimers control human trophoblast invasion. *Journal of Clinical Endocrinology and Metabolism*.

[37] McCarthy FP, Drewlo S, Kingdom J, Johns EJ, Walsh SK, Kenny LC (2011). Peroxisome proliferator-activated receptor-*γ* as a potential therapeutic target in the treatment of preeclampsia. *Hypertension*.

[38] Street ME, Seghini P, Feini S (2006). Changes in interleukin-6 and IGF system and their relationships in placenta and cord blood in newborns with fetal growth restriction compared with controls. *European Journal of Endocrinology*.

[39] Desai M, Guang Han, Ferelli M, Kallichanda N, Lane RH (2008). Programmed upregulation of adipogenic transcription factors in intrauterine growth-restricted offspring. *Reproductive Sciences*.

[40] Schaiff WT, Bildirici I, Cheong M, Chern PL, Nelson DM, Sadovsky Y (2005). Peroxisome proliferator-activated receptor-*γ* and retinoid X receptor signaling regulate fatty acid uptake by primary human placental Trophoblasts. *Journal of Clinical Endocrinology and Metabolism*.

[41] McCarthy FP, Drewlo S, English FA (2011). Evidence implicating peroxisome proliferator-activated receptor-*γ* in the pathogenesis of preeclampsia. *Hypertension*.

[42] Lappas M, Permezel M, Georgiou HM, Rice GE (2002). Regulation of proinflammatory cytokines in human gestational tissues by peroxisome proliferator-activated receptor-*γ*: effect of 15-deoxy-*δ*12,14-PGJ2 and troglitazone. *Journal of Clinical Endocrinology and Metabolism*.

[43] Vats D, Mukundan L, Odegaard JI (2006). Oxidative metabolism and PGC-1*β* attenuate macrophage-mediated inflammation. *Cell Metabolism*.

[44] Odegaard JI, Ricardo-Gonzalez RR, Goforth MH (2007). Macrophage-specific PPAR*γ* controls alternative activation and improves insulin resistance. *Nature*.

[45] Nagamatsu T, Schust DJ (2010). The contribution of macrophages to normal and pathological pregnancies. *American Journal of Reproductive Immunology*.

[46] Porcheray F, Viaud S, Rimaniol AC (2005). Macrophage activation switching: an asset for the resolution of inflammation. *Clinical and Experimental Immunology*.

[47] Martinez FO, Helming L, Gordon S (2009). Alternative activation of macrophages: an immunologic functional perspective. *Annual Review of Immunology*.

[48] Ricote M, Welch JS, Glass CK (2000). Regulation of macrophage gene expression by the peroxisome proliferator-activated receptor-*γ*. *Hormone Research*.

[49] Chawla A (2010). Control of macrophage activation and function by PPARs. *Circulation Research*.

[50] Bouhlel MA, Derudas B, Rigamonti E (2007). PPARgamma activation primes human monocytes into alternative M2 macrophages with anti-inflammatory properties. *Cell Metabolism*.

[51] Chinetti G, Fruchart JC, Staels B (2003). Peroxisome proliferator-activated receptors: new targets for the pharmacological modulation of macrophage gene expression and function. *Current Opinion in Lipidology*.

[52] Sica A, Mantovani A (2012). Macrophage plasticity and polarization: in vivo veritas. *The Journal of Clinical Investigation*.

[53] Mukundan L, Odegaard JI, Morel CR (2009). PPAR-Δ senses and orchestrates clearance of apoptotic cells to promote tolerance. *Nature Medicine*.

[54] Dong M, He J, Wang Z, Xie X, Wang H (2005). Placental imbalance of Th1- and Th2-type cytokines in preeclampsia. *Acta Obstetricia et Gynecologica Scandinavica*.

[55] Banerjee S, Smallwood A, Moorhead J (2005). Placental expression of interferon-*γ* (IFN-*γ*) and its receptor IFN-*γ*R2 fail to switch from early hypoxic to late normotensive development in preeclampsia. *Journal of Clinical Endocrinology and Metabolism*.

[56] Szekeres-Bartho J, Halasz M, Palkovics T (2009). Progesterone in pregnancy; receptor-ligand interaction and signaling pathways. *Journal of Reproductive Immunology*.

[57] Dunn SE, Bhat R, Straus DS (2010). Peroxisome proliferator-activated receptor *δ* limits the expansion of pathogenic Th cells during central nervous system autoimmunity. *Journal of Experimental Medicine*.

[58] Lindström TM, Bennett PR (2005). 15-Deoxy-Δ^12, 14^-prostaglandin J_2_ inhibits interleukin-1*β*-induced nuclear factor-*κ*B in human amnion and myometrial cells: mechanisms and implications. *Journal of Clinical Endocrinology and Metabolism*.

[59] Chiron D, Bekeredjian-Ding I, Pellat-Deceunynck C, Bataille R, Jego G (2008). Toll-like receptors: lessons to learn from normal and malignant human B cells. *Blood*.

[60] Carbone J, Gallego A, Lanio N (2009). Quantitative abnormalities of peripheral blood distinct T, B, and natural killer cell subsets and clinical findings in obstetric antiphospholipid syndrome. *Journal of Rheumatology*.

[61] Zhou CC, Ahmad S, Mi T (2008). Autoantibody from women with preeclampsia induces soluble Fms-like tyrosine kinase-1 production via angiotensin type 1 receptor and calcineurin/nuclear factor of activated T-cells signaling. *Hypertension*.

[62] Lamarca B, Wallace K, Herse F (2011). Hypertension in response to placental ischemia during pregnancy: role of B lymphocytes. *Hypertension*.

[63] Ray DM, Akbiyik F, Bernstein SH, Phipps RP (2005). CD40 engagement prevents peroxisome proliferator-activated receptor *γ* agonist-induced apoptosis of B lymphocytes and B lymphoma cells by an NF-*κ*B-dependent mechanism. *The Journal of Immunology*.

[64] Garcia-Bates TM, Baglole CJ, Bernard MP, Murant TI, Simpson-Haidaris PJ, Phipps RP (2009). Peroxisome proliferator-activated receptor *γ* ligands enhance human B cell antibody production and differentiation. *The Journal of Immunology*.

[65] Zhang J, Chen Z, Smith GN, Croy BA (2011). Natural killer cell-triggered vascular transformation: maternal care before birth?. *Cellular and Molecular Immunology*.

[66] Vacca P, Cantoni C, Vitale M (2010). Crosstalk between decidual NK and CD14^+^ myelomonocytic cells results in induction of Tregs and immunosuppression. *Proceedings of the National Academy of Sciences of the United States of America*.

[67] Lin Y, Zhong Y, Shen W (2008). TSLP-induced placental DC activation and IL-10+ NK cell expansion: comparative study based on BALB/c × C57BL/6 and NOD/SCID × C57BL/6 pregnant models. *Clinical Immunology*.

[68] González IT, Barrientos G, Freitag N (2012). Uterine NK cells are critical in shaping DC immunogenic functions compatible with pregnancy progression. *PLoS One*.

[69] French AR, Yokoyama WM (2003). Natural killer cells and viral infections. *Current Opinion in Immunology*.

[70] Zhang X, Rodriguez-Galán MC, Subleski JJ (2004). Peroxisome proliferator-activated receptor-*γ* and its ligands attenuate biologic functions of human natural killer cells. *Blood*.

[71] Gosset P, Charbonnier A-S, Delerive P (2001). Peroxisome proliferator-activated receptor *γ* activators affect the maturation of human monocyte-derived dendritic cells. *European Journal of Immunology*.

[72] Nencioni A, Grünebach F, Zobywlaski A, Denzlinger C, Brugger W, Brossart P (2002). Dendritic cell immunogenicity is regulated by peroxisome proliferator-activated receptor *γ*. *The Journal of Immunology*.

[73] Desoye G, Hauguel-De Mouzon S (2007). The human placenta in gestational diabetes mellitus: the insulin and cytokine network. *Diabetes Care*.

[74] Huppertz B (2008). Placental origins of preeclampsia: challenging the current hypothesis. *Hypertension*.

[75] Chammas MF, Nguyen TM, Li MA, Nuwayhid BS, Castro LC (2000). Expectant management of severe preterm preeclampsia: is intrauterine growth restriction an indication for immediate delivery?. *American Journal of Obstetrics and Gynecology*.

[76] Walker JJ (2000). Pre-eclampsia. *The Lancet*.

[77] Barbour LA, McCurdy CE, Hernandez TL, Kirwan JP, Catalano PM, Friedman JE (2007). Cellular mechanisms for insulin resistance in normal pregnancy and gestational diabetes. *Diabetes Care*.

[78] Hawfield A, Freedman BI (2009). Pre-eclampsia: the pivotal role of the placenta in its pathophysiology and markers for early detection. *Therapeutic Advances in Cardiovascular Disease*.

[79] Gilbert JS, Ryan MJ, Lamarca BB, Sedeek M, Murphy SR, Granger JP (2008). Pathophysiology of hypertension during preeclampsia: linking placental ischemia with endothelial dysfunction. *American Journal of Physiology*.

[80] Jawerbaum A, Capobianco E, Pustovrh C (2004). Influence of peroxisome proliferator-activated receptor *γ* activation by its endogenous ligand 15-deoxy Δ 1214 prostaglandin J_2_ on nitric oxide production in term placental tissues from diabetic women. *Molecular Human Reproduction*.

[81] Lappas M, Permezel M, Rice GE (2004). Release of proinflammatory cytokines and 8-isoprostane from placenta, adipose tissue, and skeletal muscle from normal pregnant women and women with gestational diabetes mellitus. *Journal of Clinical Endocrinology and Metabolism*.

[82] Sykes L, MacIntyre DA, Yap XJ, Ponnampalam S, Teoh TG, Bennett PR (2012). Changes in the Th1:Th2 cytokine bias in pregnancy and the effects of the anti-inflammatory cyclopentenone prostaglandin 15-deoxy-Δ^12,14^-prostaglandin J_2_. *Mediators of Inflammation*.

[83] Schaiff WT, Barak Y, Sadovsky Y (2006). The pleiotropic function of PPAR*γ* in the placenta. *Molecular and Cellular Endocrinology*.

[84] Martin H (2009). Role of PPAR-gamma in inflammation. Prospects for therapeutic intervention by food components. *Mutation Research*.

